# Association of self-reported sleep problems with morbidities and multimorbidities according to sex: National Health Survey 2019

**DOI:** 10.1590/SS2237-9622202200007.especial

**Published:** 2022-06-17

**Authors:** Margareth Guimarães Lima, Marilisa Berti de Azevedo Barros, Deborah Carvalho Malta, Lhais de Paula Barbosa Medina, Célia Landmann Szwarcwald

**Affiliations:** 1 Universidade Estadual de Campinas, Departamento de Saúde Coletiva, Campinas, SP, Brazil; 2 Universidade Federal de Minas Gerais, Escola de Enfermagem, Belo Horizonte, MG, Brazil; 3 Fundação Instituto Oswaldo Cruz, Instituto de Comunicação e Informação Científica e Tecnológica em Saúde, Rio de Janeiro, RJ, Brazil

**Keywords:** Sleep, Non-communicable Diseases, Morbidity, Health Survey, Sex Distribution

## Abstract

**Objective:**

To evaluate the association between self-reported sleep problems and the presence of non-communicable diseases (NCDs) and multimorbidity, and whether these associations differ by sex.

**Methods:**

This is a cross sectional study performed with data from the Brazilian National Health Survey, 2019. Prevalence ratios between morbidities, the number of NCDs, and the self-report of sleep problems were estimated by Poisson regression with robust variance, according to sex.

**Results:**

This study analysed data from 85,531 Brazilians age ≥ 18 years. The self-reported sleep problems were associated with all the herein studied morbidities and multimorbidities. The prevalence of sleep problems was higher in those who stated one or two (PR = 2.37; 95%CI 2.22;2.54) and three or more NCDs (PR = 4.73; 95%CI 4.37;5.11). Prevalence ratios of the association with diabetes, lung disease, mental disease, renal disease and multimorbidities were higher among males.

**Conclusion:**

NCDs significantly impacted sleep quality, with a particularly stronger association in both, males and females.

**Study contributions**

**Main results**

Strong associations of sleep problems, with herein studied diseases and multimorbidities were found. While the association between chronic non-communicable diseases (NCDs) and sleep occur in both sexes, the prevalence ratios were more significant in males.

**Implications for services**

The prevalence of chronic diseases is high and increasing, and accompanies other health issues, such as sleep problems, demanding particular attention from health services, with a special emphasis on men’s health.

**Perspectives**

Primary health care for the population with NCDs must consider the possibility of special strategies for tending to the sleep of the chronically ill people, considering its importance to health and daily life.

## Introduction

The importance of sleep duration and quality is found to be vastly evidenced.^1^ ^-^ ^3^ The daily repair processes that sleep promotes in the organism is necessary to preserve an adequate functionality of the immune, endocrine, and metabolic systems, preventing countless health problems and diseases.^1^ The main issues affecting sleep are insufficient duration, insomnia, sleep apnea, and daytime sleepiness.^4^

Studies have evidenced the association between poorer sleep quality with the presence of cardiovascular,^1^ ^,^ ^3^ metabolic,^5^ ^,^ ^6^ respiratory,^7^ ^,^ ^8^ mental^9^ ^,^ ^10^ and musculoskeletal diseases.^11^ There are indications that associations between sleep disorders and diseases might be bidirectional: while some studies detect the impact of morbidities on sleep quality,^8^ ^,^ ^12^ ^,^ ^13^ ^,^ ^14^ others suggest that insufficient sleep and sleep disturbances might lead to pathological processes and cause diseases. Evidence in this regard is more consistent with cardiovascular diseases.^2^ ^,^ ^3^ ^,^ ^15^

The tendency to appraise sleep as poor is relatively higher for women than men,^8^ ^,^ ^9^ ^,^ ^16^ much like what is observed in regard to other aspects of their health condition.^17^ The prevalence of chronic diseases is also higher in females when compared to males.^18^ However, it is acknowledged that health problems affect the male population more intensely and lethally, and there are multiple differences between both sexes in terms of health condition, illness perception, and in seeking medical attention.^17^

Many of the studies that assessed the association between sleep problems and diseases focused on the link between sleep and a particular disease,^3^ ^,^ ^7^ ^,^ ^10^ without taking into account a much larger set of chronic morbidities with different etiologies potentially associated with the event. There is little research analyzing the role of sex in this association, with some having found a higher occurrence in males^19^ ^,^ ^20^ while others having attained contradictory results,^21^ ^,^ ^22^ which may be an important factor considering the strong gender determination when it comes to health matters. In this regard, the objective of the present study was to evaluate the association between self-reported sleep problems and the presence of non-communicable diseases (NCDs) and multimorbidity, and whether these associations differ by sex.

## Methods

This was a cross-sectional, population-based study, carried out with data from the National Health Survey (*Pesquisa Nacional de Saúde* - PNS) conducted in 2019 by the Brazilian Institute of Geography and Statistics (IBGE), in partnership with the Ministry of Health. The data was accessed in March 2021, from https://bit.ly/3P5yPSa.

The PNS sample is probabilistic, representative of the Brazilian population aged 15 or older. Multi-stage cluster sampling was used, involving three stages of selection: i) census tracts, ii) households, iii) one resident from each household. Simple random sampling was used in all stages. The sample size estimate had some of the 2013 PNS’s indicators as a guideline, such as the prevalence of chronic diseases, health behaviors and others. The sampling process for the 2019 PNS is further detailed in a previous publication.^23^ In this study, specifically, the population aged 18 or over was analyzed.

The dependent or outcome variable was the sleep problems self-report, stemming from the question: *In the last two weeks, how often have you experienced sleep problems, such as difficulty to fall asleep, frequent awakening at night, or sleeping more than usual?* The response options “Not even one day” and “Less than half of the days” were aggregated and formed the category 1: “Did not have sleep problems frequently”. The answers “More than half of the days” and “Almost every day” were aggregated into category 2: “Had sleep problems frequently”. This single question was extracted from the Patient Health Questionnaire-9 (PHQ-9), a diagnostic instrument for screening individuals with major depression.^24^

The main exposure variables were: i) the self-reported medical diagnosis of NCD, which encompassed hypertension, diabetes, heart disease, stroke, asthma, other pulmonary diseases [emphysema, chronic bronchitis, or chronic obstructive pulmonary disease (COPD)], arthritis or rheumatism, spinal condition or chronic back pain, depression, other mental illnesses [anxiety disorder, panic disorder, schizophrenia, bipolar disorder, psychosis or obsessive compulsive disorder (OCD)], cancer and chronic kidney disease; ii) the number of NCDs reported: 0, 1-2, 3 or more. The presence of 3 or more morbidities was considered as multimorbidity.

The age groups were used to adjust for confounding factors (18 to 29; 30 to 49; 50 to 59; 60 to 69; ≥ 70) and sex (female; male) for adjustment and stratification.

The association between the sleep self-reports and each NCD and the number of morbidities was analyzed through Poisson regression, with robust variance, adjusted by age group and sex in the general population, and by age group in the sex-stratified analysis. The magnitude of the association was estimated by the calculation of the prevalence ratio (PR), along with the respective 95% confidence intervals (95%CI). The results were considered statistically significant once they displayed levels of statistical significance (p-values) lower than 0.05. The PR and 95%CI were also calculated according to sex stratification.

To evaluate the role of sex in the association between sleep disturbances and specific NCDs or the number or morbidities, this study used a multiplicative interaction model between sex and the regarded NCD (or the number of comorbidities), using the marginal effects of sex, age group, and the specific NCD (or the number of morbidities) as control. The variables to evaluate interaction were constructed from the multiplication of the variables of specific chronic diseases and sex, and of the number of chronic diseases and sex.

The analyses were conducted using the survey (*svy*) module in the STATA 15.0 statistics software, for the purpose of considering the effect of the study design in the data analysis.

The PNS was approved by the National Research Ethics Committee/National Health Council under Opinion No. 3,529,376, issued on August 23, 2019.

## Results

This study assessed 88,531 individuals aged 18 and older, with the mean age of 44.9 years (±17.2). Most of the population was female (53.2%) and fell within the 30 to 49 years age group (39.2%). Sleep disturbances were reported by 18.6% of the respondents, with a higher percentage for female participants (23.1%) in relation to males (13.6%) (Table 1).

**Table 1:** Distribution of age and prevalence sleep problems and NCDs^a^ studied in the Brazilian adult population according to sex, National Health Survey, 2019, Brazil

Variables	n	Prevalence (95%CI^f^)		
		Total	Female	Male
**Total**	88,531	100.0	53.2 (52.6;53.8)	46.8 (46.2;47.4)
**Age group (years)**				
18 to 2	15,394	22.1 (21.5;22.7)	21.0 (20.2;21.7)	23.4 (22.5;24.3)
30 to 49	34,752	39.2 (38.5;39.8)	39.2 (38.3;40.0)	39.2 (38.3;40.0)
50 to 59	15,657	17.1 (16.7;17.5)	16.8 (16.3;17.4)	17.5 (16.8;18.1)
60 to 69	12,555	12.2 (11.8;12.5)	12.8 (12.3;13.2)	11.5 (11.0;12.0)
≥ 70	10,173	9.4 (9.1;9.8)	10.3 (9.8;10.8)	8.5 (8.0;9.0)
Self-reported sleep problems	16,491	18.6 (18.1;19.2)	23.1 (22.4;23.8)	13.6 (13.0;14.3)
**Non-communicable diseases**				
Hypertension	23,819	25.4 (24.9;25.9)	29.2 (28.5;30.0)	21.1 (20.4;21.8)
Diabetes	7,358	8.0 (7.7;8.4)	9.0 (6.6;9.5)	6.9 (6.5;7.4)
Heart Disease	4,711	5.3 (5.0;5.6)	5.6 (5.3;6.0)	4.9 (4.6;5.3)
Stroke (CVA^b^)	1,974	2.0 (1.8;2.1)	2.0 (1.8;2.2)	2.0 (1.7;2.2)
Asthma or asthmatic bronchitis	4,294	5.3 (5.0;5.5)	6.1 (5.7;6.5)	4.4 (4.0;4.8)
Other pulmonary disease (emphysema, chronic bronchitis or COPD^c^)	1,258	1.7 (1.5;1.8)	1.7 (1.5;2.0)	1.6 (1.4;1.8)
Arthritis or rheumatism	7,199	7.6 (7.2;7.9)	11.0 (10.4;11.5)	3.7 (3.4;4.1)
Spinal condition or chronic back pain	19,029	21.6 (21.0;22.1)	24.5 (23.7;25.2)	18.3 (17.6;19.0)
Work-related musculoskeletal disorders (WRMDs^d^)	1,710	2.5 (2.3;2.8)	3.2 (2.8;3.6)	1.7 (1.5;2.0)
Depression	8,242	10.2 (9.9;10.6)	14.7 (14.1;15.4)	5.1 (4.7;5.5)
Other mental illness (anxiety disorder, panic disorder, schizophrenia, bipolar disorder, psychosis or OCD^e^)	4,792	6.5 (6.1;6.8)	8.6 (8.1;9.1)	4.1 (3.8;4.5)
Cancer	2,314	2.6 (2.4;2.7)	2.9 (2.7;3.2)	2.1 (1.9;2.4)
Chronic kidney disease	1,280	1.5 (1.3;1.6)	1.6 (1.4;1.8)	1.4 (1.2;1.6)
**Number of chronic diseases among the 13 studied**				
0	41,266	47.2 (46.5;47.8)	41.6 (40.8;42.5)	53.4 (52.6;54.3)
1 or 2	36,794	40.6 (40.0;41.2)	41.8 (41.0;42.7)	39.2 (38.4;40.1)
3 or more	10,471	12.2 (11.8;12.7)	16.5 (15.8;17.2)	7.4 (6.9;7.8)

a) NCDs: Non-communicable diseases; b) CVA: Cerebrovascular accident; c) COPD: Chronic obstructive pulmonary disease; d) WMSDs: Work-related musculoskeletal disorders; e) OCD: Obsessive compulsive disorder; f) 95%CI: 95% Confidence interval.

In Table 1, it is noted that all NCDs studied herein, and the higher number of self-reported chronic diseases, were more prevalent in females, except for stroke, chronic kidney disease and pulmonary diseases. The prevalence of self-reported sleep problems was 12.6% among the population aged 18 to 29, and 26.1%, among those aged 70 years or older (Table 2). After adjusting for age, is was found that the self-report of sleep problems was 67% higher in females and, after adjusting for sex, the prevalence increased with age, to the point of being 84% (PR = 1.84; 95%CI 1.67;2.02) and 100% (PR = 2.0; 95%CI 1.81;2.19) higher among those aged 60 to 69 years and 70 years and older, respectively, when compared with those aged 18 to 29 years (data not shown in table).

**Table 2:** Prevalence of self-reported sleep problems according to sex, age group and NCDs^a^, National Health Survey, 2019, Brazil

Variables	Prevalence (%)	95%CI^f^
**Sex**		
Male	13.6	13.0;14.3
Female	23.1	22.4;23.8
**Age group (years)**		
18 to 29	12.6	11.6;13.6
30 to 49	17.0	16.3;17.8
50 to 59	22.4	21.2;23.7
60 to 69	23.8	22.5;25.1
≥ 70	26.1	24.6;27.6
**Non-communicable diseases**		
Hypertension	27.2	26.2;28.4
Diabetes	30.2	28.4;32.2
Heart Disease	36.3	33.9;38.7
Stroke (CVA^b^)	39.0	35.1;43.1
Asthma or asthmatic bronchitis	29.2	26.8;31.7
Other pulmonary disease (emphysema, chronic bronchitis or COPD^c^)	38.7	33.6;44.1
Arthritis or rheumatism	37.6	35.5;39.8
Spinal condition or chronic back pain	31.1	29.9;32.4
Work-related musculoskeletal disorders (WRMDs^d^)	29.3	26.2;32.7
Depression	48.6	46.7;50.6
Other mental illness (anxiety disorder, panic disorder, schizophrenia, bipolar disorder, psychosis or OCD^e^)	48.5	46.1;51.0
Cancer	32.6	29.4;36.0
Chronic kidney disease	36.2	31.9;40.7
**Number of chronic diseases among the 13 studied**		
0	9.2	8.7;9.8
1 or 2	21.0	21.2;22.9
3 or more	43.8	42.1;45.7

a) NCDs: Non-communicable diseases; b) CVA: Cerebrovascular accident; c) COPD: Chronic obstructive pulmonary disease; d) WMSDs: Work-related musculoskeletal disorders; e) OCD: Obsessive compulsive disorder; f) 95%CI: 95% Confidence interval.

The prevalence of self-reported sleep problems was higher in the presence of all the diseases studied herein, with particularly stronger associations when it came to depression and other mental illnesses, back problems and pulmonary diseases. The number of diseases was also strongly associated with self-reported sleep problems, with a prevalence almost five times higher (PR = 4.73; 95%CI 4.37;5.11) in the population with three or more diseases, when compared with the ones who did not exhibit morbidities (Table 3).

**Table 3:** Prevalence ratio and 95% confidence interval (95%CI), based on the Poisson regression model, the self-reported sleep problems according to NCDs^a^, on the total and by sex, National Health Survey, 2019, Brazil

Variables	PR^f^ (95%CI^g^)			
	Total	Female	Male	Interaction (sex x NCDs^a,h^)
**Non-communicable diseases**				
Hypertension	1.50 (1.41;1.59)	1.40 (1.30;1.51)	1.48 (1.34;1.63)	
Diabetes	1.43 (1.33;1.54)	1.32 (1.21;1.43)	1.59 (1.38;1.83)	1.22 (1.04;1.42)
Heart Disease	1.76 (1.64;1.89)	1.74 (1.60;1.89)	1.76 (1.52;2.02)	
Stroke (CVA^b^)	1.76 (1.57;1.97)	1.69 (1.47;1.95)	1.95 (1.63;2.32)	
Asthma or asthmatic bronchitis	1.67 (1.53;1.83)	1.62 (1.47;1.78)	1.56 (1.28;1.90)	
Other pulmonary disease (emphysema, chronic bronchitis or COPD^c^)	1.96 (1.70;2.26)	1.76 (1.44;2.14)	2.34 (1.93;2.84)	1.34 (1.02;1.76)
Arthritis or rheumatism	1.89 (1.76;2.02)	1.68 (1.57;1.81)	1.81 (1.54;2.12)	
Spinal condition or chronic back pain	1.89 (1.79;1.99)	1.79 (1.68;1.91)	1.87 (1.70;2.05)	
Work-related musculoskeletal disorders (WRMD^d^)	1.52 (1.35;1.70)	1.35 (1.17;1.55)	1.64 (1.30;2.07)	
Depression	3.07 (2.91;3.23)	2.61 (2.46;2.76)	3.58 (3.24;3.95)	1.38 (1.23;1.54)
Other mental illness (anxiety disorder, panic disorder, schizophrenia, bipolar disorder, psychosis or OCD^e^)	3.04 (2.87;3.22)	2.54 (2.37;2.72)	3.79 (3.41;4.22)	1.49 (1.31;1.69)
Cancer	1.48 (1.33;1.64)	1.38 (1.22;1.57)	1.61 (1.33;1.90)	
Chronic kidney disease	1.77 (1.56;2.00)	1.52 (1.31;1.77)	2.40 (1.81;2.77)	1.49 (1.15;1.93)
**Number of chronic diseases among the 13 studied**				
0	1.00	1.00	1.00	-
1 or 2	2.37 (2.22;2.54)	2.18 (2.00;2.38)	2.48 (2.22;2.77)	
3 or more	4.73 (4.37;5.11)	3.99 (3.62;4.41)	5.07 (4.44;5.77)	1.26 (1.09;1.46)

a) NCDs: Non-communicable diseases; b) CVA: Cerebrovascular accident; c) COPD: Chronic obstructive pulmonary disease; d) WMSDs: Work-related musculoskeletal disorders; e) OCD: Obsessive compulsive disorder; f) PR: Prevalence ratios adjusted by age; g) 95%CI: 95% Confidence interval; h) Regression model with interaction terms adjusted by specific NCD (or number of comorbidities), age group and sex (reference category female).

In terms of the association between self-reported sleep problems, the analyses revealed significant interactions between sex and the presence of diabetes, pulmonary diseases, depression, other mental illnesses and chronic kidney disease, suggesting that there is an interaction between sex and these NCDs, towards the increased prevalence of self-reported sleep problems (Table 3).

## Discussion

The main results of this study were the strong associations found between self-reported sleep problems and all the diseases studied herein and number of morbidities. The prevalence of these problems was roughly twice more prominent in individuals that reported the diagnosis of one or two diseases, and approximately four times higher in those with three morbidities or more, in contrast to the ones who reported not having the studied diseases. The prevalence of NCDs, in general, was higher among females and, while the associations between NCDs and sleep have occurred for both sexes, the prevalence ratios were more expressive for males.

The results found in regard to the correlation between poorer sleep quality and NCDs are similar to the ones already described.^2^ ^,^ ^6^ Possible biological explanations for the associations between vascular problems and poorer sleep quality are the altered levels of leptin and ghrelin, making way for obesity and hormonal changes, such as increased cortisol levels resulting from inadequate or insufficient sleep. Sleep deprivation may also increase stress response, altering blood pressure and increasing cardiovascular risk factors.^3^ According to a meta-analysis, deteriorating sleep quality is also associated with the increase of glycated hemoglobin in people with type 1 diabetes.^5^ Good quality sleep appears to be importantly associated with glycaemic control in adults with type 2 diabetes, as highlighted by Zhu et al. (2017).^6^ The evidences indicate the need for increased care when it comes to the sleep quality of individuals who display these morbidities.

Respiratory diseases, COPD above all, were also frequently associated with the self-report of sleep disturbances in this study, corroborating the previous study that indicated lower sleep efficiency among people with COPD, who had strong symptoms of insomnia.^7^ Among the factors that are possibly responsible for that are nocturnal hypoxemia, the presence of cough and expectoration.^8^

The association between poorer sleep quality and back and musculoskeletal problems also stands out in the present study. Sleep problems have been associated with chronic pain,^11^ particularly those prevenient from musculoskeletal disorders.^12^ The inflammatory processes resulting from the insufficient duration or poor quality of sleep may be a triggering factor for chronic pain, but the matters regarding pain and sleep appear to act in a vicious cycle, in which sleep deprivation might lead to pain and, likewise, the pain itself might give rise to poor sleep.^12^ The decrease in physical and functional activity, the discomfort caused by the pain and sleep position could be possible explanations for how pain impacts sleep quality. Moreover, pain may impair mental health, leading to depression or anxiety, which, indirectly, adversely affects sleep.^11^

The association between emotional and mental issues and sleep problems can be observed in this study as well as in other research.^9^ ^,^ ^16^ ^,^ ^20^ A meta-analysis conducted with prospective studies found that the chance of depression in the presence of insomnia is two times higher.^10^ Studies carried out in Brazil during the COVID-19 Pandemic have reinforced this significant association.^16^ In this research, depression and other mental illnesses have shown to be more strongly associated with sleep problems when compared to the other diseases and conditions studied, denoting a need for monitoring the sleep quality of the population suffering from depression and other mental illnesses.

Although the relation between cancer and sleep problems may come about in different ways, due to the heterogeneity in the types of neoplasms and, thus, complicate the understanding of cause and effect, it is argued that the tumors interrupt normal homeostasis, overburdening the organism, particularly the sleep structure.^25^ In addition to this, the effects of the disease on mental health, the impact of the treatment and the negative impacts sustained in the lifestyle, can all lead to sleep disturbances. In agreement with the literature, the present study suggests a association between self-reported sleep problems and chronic kidney disease.^26^ A study carried out with data from the National Health and Nutrition Examination Survey, in the United States, encountered a higher prevalence of sleep medication usage, restless leg syndrome and nocturia in individuals with renal dysfunction, when compared to those who did not have such alteration.^26^

The occurrence of only one or two diseases was associated, in great magnitude, with sleep problems, but this study draws attention to the multimorbidities, considering that the presence of three morbidities or more represented an increase over four times higher in the report of sleep problems. In Campinas’s Municipal Health Survey (ISACamp 2014/15), the prevalence of poor sleep was 94% higher among the population who reported five or more morbidities.^9^ Using data from the European Health Examination Survey 2013-2015, in Luxemburg, the authors found that the chance for insufficient sleep was six times higher (OR = 6.79) when three or more chronic diseases were reported.^27^ Possible rationale for the strong correlation between poor sleep and the amount of morbidities are the physiological changes that occur due to the associated morbidities, polypharmacy and impaired mental health. The study by Lima et al. (2021)^16^ showed that mental health is responsible for 45% of the associations between multimorbidities and worsened sleep quality.

The prevalence of self-reports for most of the chronic diseases and multimorbidities studied herein, was higher among females, corroborating previous evidence^17^ ^,^ ^18^ in the same manner that the self-report of sleep problems was also higher in this group.^8^ ^,^ ^9^ ^,^ ^16^ ^,^ ^20^ The female population tends to have a higher prevalence of diseases, health complaints, of self-assessing their health status as poor, and of worse diagnosed and self-reported emotional conditions.^17^ ^,^ ^18^ These differences may be due to the female population having a broader perception of health issues, being more prone to seeking treatment for health concerns and due to their experiences with caring for relatives, in comparison to males.^17^ On the other hand, men subject themselves to worse health behaviors and are victims of a higher occurrence of early mortality.^17^ It is plausible that a culture that accepts and favors a perception of higher fragility in women, demands more restrained attitudes and behaviors of men when it comes to complaints about pain, discomfort, distress and health, which may lead them to underestimate sleep problems.

There is a lack of population-based research addressing the relation between NCDs and sleep, analyzed according to sex, particularly in Brazil. The present study revealed significant interactions between sex and some of the NCDs studied. According to the presented results and after performing adjustments, although female individuals report worse health conditions and worse sleep quality, the associations between these factors showcased greater magnitude within the male population. These results are not in concordance with those attained by Lao et al. (2018).^3^ However, a study that used the national primary care database, containing 1,275,461 Chinese adults, found that obstructive sleep apnea was more prevalent in men with type 1 and 2 diabetes than in women.^19^ Among Norwegian teenagers, sleep deprivation and insomnia were strongly associated with depression, with a grander magnitude in boys. Although the authors assume that the mechanisms for these differences are still unknown, they briefly discuss the results in the lighter of gender paradox.^20^

Regarding respiratory diseases, two studies that assessed sex interactions in sleep disturbances exhibited contradictory results. A case-control study with elders revealed an association between sleep disturbances and COPD in men, but in women the differences were not significant.^21^ In Norway, an association between respiratory diseases and sleep apnea was observed only in women.^22^

In this research, the presence of an isolated chronic disease was not analyzed, therefore it is possible that a specific disease could be accompanied by other morbidities, seeing that 12.9% of the assessed population reported having three or more morbidities. However, the diseases tend to be amassed in patterns^28^ and the results may, partially, translate the effects of the related pattern to the disease in question.

Some limitations of this study must be considered. The sleep problems were self-reported, however, a study conducted with samples of 1,998 individuals suggested that sleep self-reports are strongly associated with sleep complaints.^9^ Although there are specific and validated instruments to assess sleep quality, other studies have also been adopting unique self-assessment questions.^29^ The information about the non-communicable diseases was self-reported, although the question had inquired about the medical diagnosis of the morbidity. These reports may introduce memory and information biases, and might lead to underestimation of the estimates encountered. It is also worth considering the study’s cross-sectional design, which does not allow for evaluating cause and effect, therefore, it is possible that the chronic diseases are impacting the population’s sleep or, contrarily, that the sleep problems are leading to the occurrence of these diseases.

The present study fills a gap by examining the association between self-reported sleep problems and a group of chronic morbidities and multimorbidities, analyzing the modifying role of sex in these associations. The analysis was carried out with an ample sample, that allowed the assessment of even rarer events, more difficult to access in a population basis. On another hand, it is certain that big sample sizes are prone to encountering significant differences in statistical tests, even when the associations are weak. The results here showcased should, therefore, be examined with this limitation in mind.

The sleep of people with chronic diseases, particularly those that display multimorbidities, is cause for attention. An important finding was the interaction between sex and NCDs in the increase of the prevalence of sleep problems, showing that the associations were significantly more expressive in the male population. Therefore, this article calls attention to the health care of this population, as well as to the need for promoting preventive measures focused on male health, and the upkeep of Brazil’s National Comprehensive Health Care Policy for Men (*Política Nacional de Atenção Integral à Saúde do Homem*),^30^ taking into account this group’s health specificities. 

## References

[1] Faraut B, Boudjeltia KZ, Vanhamme L, Kerkhofs M (2012). Immune, inflammatory and cardiovascular consequences of sleep
restriction and recovery. Sleep Med Rev.

[2] Kwork CS, Kontopantelis E, Kuligowski G, Gray M, Muhyaldeen A, Gale CP (2018). Self-reported sleep duration and quality and cardiovascular
disease and mortality: a dose-response meta-analysis. J Am Heart Assoc.

[3] Lao XQ, Liu X, Deng HB, Chan TC, Ho KF, Wang F (2018). Sleep quality, sleep duration, and the risk of coronary heart
disease: a prospective cohort study with 60,586 adults. J Clin Sleep Med.

[4] Chokroverty S (2010). Overview of sleep & sleep disorders. Indian J Med Res.

[5] Reutrakul S, Thakkinstian A, Anothaisintawee T, Chontong S, Borel AL, Perfect MM (2016). Sleep characteristics in type 1 diabetes and associations with
glycemic control: systematic review and meta-analysis. Sleep Med.

[6] Zhu B, Hershberger PE, Kapella MC, Fritschi C (2017). The relationship between sleep disturbance and glycaemic control
in adults with type 2 diabetes: an integrative review. J Clin Nurs.

[7] Valipour A, Lavie P, Lothaller H, Mikulic I, Burghuber OC (2011). Sleep profile and symptoms of sleep disorders in patients with
stable mild to moderate chronic obstructive pulmonary
disease. Sleep Med.

[8] Budhiraja R, Siddiqi TA, Quan SF (2015). Sleep disorders in chronic obstructive pulmonary disease:
etiology, impact, and management. J Clin Sleep Med.

[9] Barros MBA, Lima MG, Ceolim MF, Zancanella E, Cardoso TAMO (2019). Quality of sleep, health and well-being in a population-based
study. Rev Saude Publica.

[10] Baglioni C, Battagliese G, Feige B, Spiegelhalder K, Nissen C, Voderholzer U (2011). Insomnia as a predictor of depression: a meta-analytic evaluation
of longitudinal epidemiological studies. J Affect Disord.

[11] Burgess HJ, Burns JW, Buvanendran A, Gupta R, Chont M, Kennedy M (2019). Associations between sleep disturbance and chronic pain intensity
and function: a test of direct and indirect pathways. Clin J Pain.

[12] Lavigne GJ, Nashed A, Manzini C, Carra MC (2011). Does sleep differ among patients with common musculoskeletal pain
disorders?. Curr Rheumatol Rep.

[13] Miner B, Kryger MH (2017). Sleep in the aging population. Sleep Med Clin.

[14] Garcia AD (2008). The effect of chronic disorders on sleep in the
elderly. Clin Geriatr Med.

[15] Troxel WM, Buysse DJ, Mathews KA, Kip KE, Strollo PJ, Hall M (2010). Sleep symptoms predict the development of the metabolic
syndrome. Sleep.

[16] Lima MG, Malta DC, Werneck AO, Szwarcwald CL, Souza DBO, Gomes CS (2021). Effect of chronic non-communicable diseases (CNCDs) on the sleep
of Brazilians during the COVID-19 pandemic. Sleep Med.

[17] Bastos TF, Canesqui AM, Barros MBA (2015). “Healthy Men” and high mortality: contributions from a
population-based study for the gender paradox discussion. PLoS One.

[18] Rzewuska M, Azevedo-Marques JM, Coxon D, Zanetti ML, Zanetti ACG, Franco LJ (2017). Epidemiology of multimorbidity within the Brazilian adult general
population: evidence from the 2013 National Health Survey (PNS
2013). PLoS One.

[19] Feher M, Hinton W, Munro N, Lusignan S (2019). Obstructive sleep apnoea in Type 2 diabetes mellitus: increased
risk for overweight as well as obese people included in a national primary
care database analysis. Diabet Med.

[20] Sivertsen B, Harvey AG, Lundervold AJ, Hysing M (2014). Sleep problems and depression in adolescence: results from a
large population-based study of Norwegian adolescents aged 16-18
years. Eur Child Adolesc Psychiatry.

[21] Theorell-Haglöw J, Ólafsdóttir IS, Benediktsdóttir B, Gíslason T, Lindberg E, Janson C (2016). Sex differences in reported and objectively measured sleep in
COPD. Int J Chron Obstruct Pulmon Dis.

[22] Jonassen TM, Eagn TM, Bjorvatn B, Lehmann S (2018). Associations between obstructive lung disease and symptoms of
obstructive sleep apnoea in a general population. Clin Respir J.

[23] Stopa SR, Szwarcwald CL, Oliveira MM, Gouvea ECDP, Vieira MLFP, Freitas MPS (2020). Pesquisa Nacional de Saúde 2019: histórico, métodos e
perspectivas. Epidemiol Serv Saude.

[24] Santos IS, Tavares BF, Munhoz TN, Almeida LSP, Silva NTB, Tams BD (2013). Sensibilidade e especificidade do Patient Health Questionnaire-9
(PHQ-9) entre adultos da população geral Cad. Saude Publica.

[25] Walker WH, Borniger JC (2019). Molecular Mechanisms of Cancer-Induced Sleep
Disruption. Int J Mol Sci.

[26] Plantinga L, Lee K, Inker LA, Saran R, Yee J, Gillespie B (2011). Association of sleep-related problems with CKD in the United
States, 2005-2008. Am J Kidney Dis.

[27] Ruiz-Castell M, Makovski TT, Bocquet V, Stranges S (2019). Sleep duration and multimorbidity in Luxembourg: results from the
European Health Examination Survey in Luxembourg, 2013-2015. BMJ Open.

[28] Nunes BP, Batista SRR, Andrade FB, Souza PRB, Lima-Costa MF, Facchini LA (2018). Multimorbidade em indivíduos com 50 anos ou mais de idade:
ELSI-Brasil. Rev Saude Publica.

[29] Ohayon MM, Zulley J (2001). Correlates of global sleep dissatisfaction in the German
population. Sleep.

[30] Brasil. Ministério da Saúde. Secretaria de Atenção à Saúde.
Departamento de Ações Programáticas Estratégicas (2008). Política Nacional de Atenção Integral à Saúde do Homem. Princípios e
Diretrizes.

